# Transition to Labor Market among Young Adults with Serious Mental Illness

**DOI:** 10.3390/ijerph19084532

**Published:** 2022-04-09

**Authors:** Inbal Boaz, Eynat Ben Ari, Lena Lipskaya-Velikovsky, Navah Z. Ratzon

**Affiliations:** 1 Occupational Therapy Department, School of Health Professions, Sackler Faculty of Medicine, Tel Aviv University, Ramat Aviv, Tel Aviv 69978, Israel; inbalb@s-tov.org.il (I.B.); eynatbenari@gmail.com (E.B.A.); navah@tauex.tau.ac.il (N.Z.R.); 2 Almog, Holon 58000, Israel; 3 School of Occupational Therapy, Faculty of Medicine, The Hebrew University, Jerusalem 91905, Israel

**Keywords:** vocational rehabilitation, supported employment, cognition, motor functioning, instrumental activities of daily living

## Abstract

Background: The research on job attainment and retention among young adults with serious mental illness (SMI) is limited. The objective of this study was to investigate the contributions of emotional, cognitive, motor, demographic, and work-related factors to the transition into supported employment (SE) and retention. Methods: This cross-sectional study included young adults with SMI involved in prevocational (*N* = 21) services or those who have transferred to SE (*N* = 21) following prevocational services. Work-related self-efficacy, executive functions, and motor skills were approached with standard and well-established tools. Results: There was a significant difference between groups in most dimensions of work-related self-efficacy, job history and experience, cognitive strategies, and general independence in daily life. The multivariate analysis demonstrates that holding a profession, experiencing self-efficacy in general work skills, cognitive strategies, and independence in living situations explained the between-group differences (χ^2^(4) = 34.62, *p* < 0.001; correct classification–90.2%). Conclusions: The study identifies the factors contributing to a sustainable transition to employment among young adults with SMI, suggesting the importance of a comprehensive approach to address a range of personal factors in an integrative way. The augmentation of prevocational training with continued employment support may be beneficial to meet the unique needs of young adults with SMI.

## 1. Introduction

Employment is an important aspect of everyday life, contributing to the health and recovery of those with serious mental illness (SMI) [1,2,3]. SMI encompasses mental, behavioral, or emotional disorders resulting in serious functional impairment, which substantially interferes with or limits one or more major life activities [4]. Employment provides people with meaning, identity, and belonging, and offers opportunities for financial stability, social inclusion, a structured lifestyle, and an experience of value for others and society as a whole. In contrast, unemployment is associated with poor health parameters, financial stress, and social isolation [3,5,6,7]. For decades, there have been significant efforts to support people with SMI to obtain valuable and sustainable employment, which lead to the development of various approaches, programs, and services [1,8,9]. Moreover, an international standard for vocational rehabilitation—the Individual Placement and Support (IPS) program—was accepted [1,8,9,10]. Still, the recent literature demonstrates that people with SMI continue to be under-represented in the labor market due to difficulty in either obtaining or holding a job [3,9]. Indeed, although the IPS programs, which are guided by the “place and train” approach, were found to be more effective than additional approaches, such as “train then place” (prevocational training), and are widely implemented all over the world, replacing in practice and research programs, their effectiveness is limited [9,10,11,12]. The research reveals that IPS did not enable employment for many participants and job tenure obtained through IPS is still relatively short [9,10,11,12]. These findings reveal a need for further reconsideration of vocational rehabilitation, which should be firstly based on comprehensive research on the factors enabling sustainable employment.

Young adults with a higher incidence of mental health conditions among all age groups and a longer expected period in the labor market may be a particularly vulnerable population for unemployment [13,14]. Indeed, the findings on high unemployment rates are even more prominent for this age group [15]. Previous research on factors contributing to employment demonstrates that young adults with SMI may have personal barriers that are known in other age groups. These barriers comprise psychiatric symptoms, including cognitive impairment (e.g., memory, problem solving, and executive functions), side effects of medication, a low sense of self-efficacy (defined as an individual’s confidence that they can carry out the behaviors or tasks required to achieve a particular outcome [16]), and inferior motor skills [17,18,19,20]. However, some of these barriers, such as self-efficacy, self-awareness, the experience of autonomy, and capability in work tasks, are of particular importance and may have an even higher impact among young adults [13]. Moreover, this age group was reported to experience unique difficulties for integration into the labor force due to limited work history and job-related experience and interference in the processes of education and skills acquisition [13,14]. However, little research has been conducted to investigate the interplay between the various factors affecting employment outcomes [13]. Thus, the complex job-related needs of young adults with SMI are still mostly unmet [13]. In order to design relevant services for this age group, comprehensive research on the factors enabling the sustainable integration of this population into the labor market is needed. 

The research on enablers for employment should consider the features of local context. The local context of each specific country influences employment through a range of economic, social, and political aspects, such as general unemployment rates, levels of disability benefits compensation, educational and job market opportunities, labor unionization activity, regulations for employment protection, policies for the mandatory integration of people with SMI into the workforce, etc. [9,21]. For example, it was found that the level of effectiveness of IPS was reduced in countries with stronger employment protection legislation, higher integration efforts with respect to people with SMI, and higher disability benefits [9,22]. While most of the research in the field of vocational rehabilitation was conducted in Western countries, such as the United States, UK, Canada, and Australia, the findings indicate a need to investigate the factors contributing to sustainable employment in each specific context to address a local reality. There is a range of vocational rehabilitation programs in Israel, which are provided within the Community Mental Health Rehabilitation Law. However, to date, little research has been conducted in Israel to investigate the factors contributing to employment outcomes among young adults with SMI [23]. Such research is of importance given our limited understanding of the phenomenon. For example, the patterns of actual participation in rehabilitation services were reported to be different in Israel compared with other Western countries. Even though a range of supported employment programs exist, more people use the services of sheltered workshops [24]. In addition, the costs of non-employment of people with SMI in Israel are high in terms of the loss in Gross Domestic Product and the investment in vocational rehabilitation, yielding a low level of actual employment [25]. 

Given the unique issues among young adults with SMI regarding employment and the little research in the field in Israel, the study subject was to comprehensively investigate the enablers for sustainable integration into the labor market in this population. Our aims were as follows: (1) to reveal the differences in emotional, cognitive, and motor factors, as well as demographic and work-related history between those receiving prevocational services and those who transfer to supported employment with retention following prevocational services; (2) to build a multi-factor model to explain sustained employment. Based on the literature review, the premise was that the differences will be found between the groups in all the investigated factors. However, we hypothesized that the most contributing factors to the group distinction will be the cognitive and emotional factors. 

## 2. Materials and Methods

### 2.1. Participants and Settings

Forty-two young adults who were diagnosed with either schizophrenia, bipolar disorder, major depression, anxiety, or personality disorder (DSM 5) [26] (all of these diagnoses meet the NIMH diagnosis of SMI [4]) (26 male, 16 female) and received vocational rehabilitation services for young adults completed this cross-sectional study. The vocational rehabilitation for young adults with SMI comprises two stages. First, the prevocational program developed for young adults was dedicated to meet their unique employment needs. 

Forty-two young adults who were diagnosed with either schizophrenia, bipolar disorder, major depression, anxiety, or personality disorder (DSM 5) [26] (all of these diagnoses meet the NIMH diagnosis of SMI [4]) (26 male, 16 female) completed this cross-sectional, naturalistic, comparative study. Based on the inclusion criteria, all the study participants were aged 20–30, received psychiatric medication as prescribed according to their report, were eligible for rehabilitation services after receiving 40% or more of benefits due to a psychiatric disability according to the Israeli National Insurance Institute, were involved in the vocational rehabilitation services, and reported a sufficient level of local language literacy to complete the evaluations. The exclusion criteria were developmental or acquired neurological conditions and/or physical disability co-occurring with the psychiatric diagnosis. The sample size was calculated based on the previous study on the differences in cognitive functioning between unemployed people with SMI who wanted to work and people with SMI who received SE [27]. We based the calculation on between-group differences in the TMT-B score as a measure of executive functions. With the significance level of 0.05 and power of 0.8, the sample size was found to be *N* = 21. The study received ethical approval from the Institutional Review Board. Informed written consent was obtained from all the participants after providing an explanation on the study aims and procedures. 

The participants were recruited through convenience sampling from the only vocational rehabilitation program dedicated to young adults with SMI. The program comprised two stages. The first stage was the prevocational program developed for young adults that was devoted to meet their unique employment needs. The program addressed general work skills (such as curriculum vitae writing), identification of interests and needs in the workplace, skills of socializing with coworkers, strategies for stress management and problem solving, the ability to meet deadlines and keep times, and productivity rate, etc. The program was managed through group and individual sessions that included discussions, role playing, and the development of individualized plans for the practicing of personal issues, such as skills training within workplace simulations. The program curriculum took place over 5 days, spanning 4 h per day. The second stage of the vocational rehabilitation for young adults was a supported employment (SE) program, which was managed according to the standard principles of IPS with local adaptations as to the time for placement, negotiations between client preferences, job market realities, etc. The SE services were provided by eight regional agencies from geographical areas that work collaboratively with the prevocational program. All the SE group participants completed the prevocational program, transferred to the labor market, and received continuous individual support at their workplace after starting the job. 

The SE group (*N* = 21), aged 20 to 30 (M = 26.33, SD = 2.86), consisted of participants who completed prevocational training for young adults and were actually working in competitive jobs. The second group encompassed people who had been currently receiving prevocational training for young adults (the same service) (*N* = 21), aged 20 to 30 (M = 25.85, SD = 2.78). The groups were matched by age and gender with no significant differences between groups (U = 58.5, *p* > 0.05; χ^2^ = 0.188, *p* > 0.05, respectively). In addition, there were no differences found in illness duration between groups (Table 1). The details of participants’ characteristics by groups are shown in Table 1 and Table 2. 

### 2.2. Measures 

#### 2.2.1. Demographic Questionnaire 

The demographic questionnaire included questions on personal information (e.g., marital status and housing situation), participant employment history (including if they had acquired a profession before beginning the vocational rehabilitation), occupational history, and current degree of independence in activities of daily living (ADL), instrumental activities of daily living (IADL), and housekeeping, based on self-experience. For those who worked, their overall number of working hours per week was recorded.

#### 2.2.2. The Work-Related Self-Efficacy Scale (WSS-37) 

The WSS-37 [28] was used to examine participants’ level of confidence in their ability to complete activities or tasks related to obtaining and maintaining employment. The inventory yielded the following scores: career planning skills, job securing skills, work-related social skills, general work skills, and a work-related self-efficacy composite score. Participants were asked to rate their degree of confidence from 0% (no confidence) to 100% (total confidence) in 10% confidence increments. The test is well established in the field with reported internal consistency (the alpha coefficients for the four separate domains of the instrument ranging from 0.85 to 0.94, and 0.96 for the entire scale), test-retest reliability (r = 0.98), and face validity. 

#### 2.2.3. Zoo Map 

The Zoo Map [29] test (both parts) from the Behavioral Assessment of the Dysexecutive Syndrome battery was used in this study to estimate the effects of dysexecutive syndrome. The Zoo test examines the ability to plan, self-monitor, problem-solve, shift, attend, and use working memory. The Zoo test score is based on the sum of correct steps completed. In addition, we measured the test completion time. Test-retest reliability (r = 0.34), construct validity (0.41 ≤ r ≤ 0.63), and discriminant validity (U = 420) were previously demonstrated [29]. 

#### 2.2.4. Motor Skills Assessments

The Purdue Pegboard Test (PPT) [30] was used to measure manual dexterity, testing gross and fine movements of hands, fingers, arms, and fingertip dexterity. The score is based on the sum of pins that were inserted in a given time for each hand and for both hands. The PPT psychometric properties are well established, including test-retest reliability (0.37 < r < 0.82), construct validity (0.52 < r < 0.78), and internal consistency (0.41 < Cronbach α < 0.50) [31]. This is a widely used test in a range of health conditions, including mental health [32,33]. 

The dynamic strength section of the Physical Work Performance Evaluation (PWPE) [34] was used as the lifting dynamic strength evaluation. This test evaluates the ability to lift a box from the floor to pelvis height. This task has been shown to be the best predictor of dynamic strength [35]. The variable that was examined was the safe maximum weight (in kilograms) for the task. The resulting score was the total weight (in kilograms) that the participant was able to lift. The test has construct validity and was found to be a strong predictor of returning to work [35,36]. 

### 2.3. Procedure 

The participants were recruited using convenience sampling from a prevocational training program for young adults and the SE community program that was provided by eight local regional agencies, following the young adults’ prevocational program. The potential participants were approached via their care coordinator. Those who agreed to participate in the study met with a researcher (I.B.) to receive additional explanation on the study procedures and provide informed consent. Following this, the research tools were administrated over one to two sessions, according to personal preferences for time and place. The questionnaires and cognitive evaluations were followed by motor assessments. Fifty people were eligible to participate in the study according to inclusion and exclusion criteria and were approached; 42 of them consented to take part in the study and completed the research procedures.

### 2.4. Statistical Analyses 

SPSS version 27 was used to analyze the data. The level of significance was set at 0.05 for all statistical tests. A *t*-test and a Mann–Whitney test for independent samples were calculated according to the distribution of the variables to study between-group differences in the study variables of self-efficacy, motor and cognitive skills, and demographic data. The chi-square test was used for categorical variables. The logistic regression analysis was conducted in order to study the multivariate correlations between the dependent (SE/prevocational training) and independent variables, which were selected based on the level of significance in the preliminary analysis. Motor skills measurements were excluded from the regression analysis as there were no significant differences between the groups. 

## 3. Results

### 3.1. Between-Groups Differences 

Illness and work-related history among the groups were compared. The young adults who were employed had three times more years of accumulated work experience before beginning vocational rehabilitation than the prevocational training group (Table 1), who frequently lived outside the parental home, acquired a profession prior to rehabilitation, and needed less help with IADL (Table 2).

As shown in Table 3, work-related self-efficacy was significantly higher among the workers in the dimensions of career-planning skills, job-securing skills, and general work skills, but not in social work skills. No significant differences were found between the groups in the measures of motor and cognitive skills, except for a difference in total performance time in part 2 of the Zoo test, which was longer among the workers. 

### 3.2. Explanatory Analysis 

The model for the multivariate explanation of the vocational rehabilitation stage includes the following: a general work skills subscale of a self-efficacy questionnaire, having a profession, living situation, and time for completion of the Zoo test. The logistic regression model was found to be significant (χ^2^(4) = 34.62, *p* < 0.001), explaining 76% of the between-groups differences and classifying, in a correct way, 90.2% of the participants. The coefficients of all the independent variables in the model were significant. The likelihood of transitioning to work with retention among young adults living with SMI is 1.2 times higher for those with higher self-efficacy on general work skills, 1.02 times higher for those who perform slowly on version 2 of the Zoo test, 290 times higher for those who had a profession, and 0.02 lower for those who live in the parental home (OR) (Table 4). 

## 4. Discussion

This study comprehensively investigates the enablers for sustainable integration into the labor market among young adults with SMI in Israel, thus, meeting an urgent call to re-examine mental health care for this population to address their unique needs [13]. To meet this goal, we investigated the differences in work-related self-efficacy, motor skills, cognitive skills, and personal and work history between young adults living with SMI who transfer into employment with retention and those who participated in the prevocational training. The factors that were found to contribute to the integration in the labor market and retention were holding a profession, experience of self-efficacy in general work skills, usage of adequate cognitive strategies in complex tasks, and general independence in a range of everyday activities. These findings are of importance for the understanding of the complex interplay between a range of factors relevant to employment in this population, and determining potential venues to promote the vocational outcomes among young adults with SMI. 

The study results demonstrate that following the program, which includes prevocational training and following SE, an average tenure in the workplace was longer than the previously reported tenure average rate in the SMI population (2.5–6.6 months) [3,37]. The disparity in the results may be explained by the different age groups of the participants in the previous studies in comparison to this study population. However, the results may be ascribed to the advantages of long-term support in employment as was previously delineated [13] and to the contribution of the prevocational training program for the tenure among young adults with SMI. It may be assumed that prevocational training and continuing SE are effective for young adults, targeting their unique needs. Interestingly, previous studies had demonstrated the contribution of augmented therapies with SE in general and with IPS in particular to improve the vocational outcomes (e.g., greater number of hours to work and job retention) for a range of ages [1,12,37]. There is a range of interventions that are commonly augmented with IPS, such as cognitive behavioral therapy and neurocognitive interventions, all of which address work-related specific issues [1,13]. The SE group of this study may share the benefits of the augmented program involving the combination of prevocational programs that address a range of work-related issues and skills through various types of intervention, as was provided in this study with SE, further explaining the findings on higher job retention rate. 

The role of cognitive skills and strategies in vocational outcomes was previously widely demonstrated (e.g., [18,19,20]). Indeed, the study demonstrates that strategies for coping with a complex cognitive task are relevant to sustainable integration into the labor market. However, interestingly, it was found that working young adults with SMI are characterized by taking a longer time to complete recurrent cognitive tasks which require action under complex guidelines. At first glance, the results may seem confusing, since the findings may be interpreted as inferior to the prevocational training group. Still, we should understand them in light of vocational outcomes. Taking a longer time to complete a recurrent task, after facing complexity at first with little success, may indicate a lower level of impulsivity and more adaptive coping strategies. Previous studies indicate that impulsivity is one of the cognitive difficulties frequently reported among people with SMI, which leads to difficulty in learning and reasoning and has a direct correlation with poorer work habits, relatively low job quality, and fewer working hours [38,39]. The findings suggest that people who were integrated in work were less impulsive and had more effective strategies of cognitive control to cope with a complex task for the second time. 

Holding a profession before SE and having previous work experience were found to be factors distinguishing between the groups and of particular importance for sustainable integration into the workplace. These findings may further support the previous notion of the importance of well-established cognitive and coping strategies for sustainable employment [40]. However, the findings bring some new insights on the importance of prerequisites for employment among young adults through pathways other than cognitive ones. For example, people with a profession and work experience may have more opportunities for job attainment or may be seen by an employer as more reliable. Moreover, there are benefits on a personal level, as having a profession and work experience may contribute to self-esteem and self-efficacy, which will be addressed below. Since work experience and profession holding contribute to the potential of employment with retention through multiple mechanisms, investment in profession acquisition and development may be seen as a valuable target among young adults with SMI. Even though the current evidence indicates an advantage of the “place and train” approach (e.g., IPS) to vocational rehabilitation, it is reasonable to assume that the “train then place” approach has some advantages in this context [1]. Prevocational services designed to support the development and practice of personally- and community-valued work-related skills and knowledge may help overcome the challenges posed by SMI [1,41,42]. 

Significant differences were found between the SE group and the prevocational training group in most measures of work-related self-efficacy. Moreover, following the previous research, the experience of competence in general work skills and the capability of managing job tasks and chores were detected to be factors expanding the chance of sustainable employment. The relationship between work-related self-efficacy and work integration has been previously depicted in the general population; it is even more imperative among adults with SMI, suggesting that it is a significant enabling factor for integration into the labor market [11,13,43]. Still, the findings of this study on the contribution of self-efficacy to the transition into the labor market require some caution. Self-efficacy is of a dynamic quality; the experience of success in facing job requirements might further contribute to the building of self-efficacy, while difficulties in overcoming obstacles in the workplace may reduce self-efficacy [13,37]. This cross-sectional study is limited in its ability to detect the nature of interplay between self-efficacy and the transition into employment, since it may be assumed that the SE group participants who integrated into employment have the potential for proliferation of their self-efficacy. However, in both cases and given the previous findings, the study supports the importance of addressing work self-efficacy in the early stages of adulthood. 

An additional interesting finding of this study is the connection between independence in one’s living situation to employment status. Independent living involves performance of IADL, which requires multiple cognitive, interpersonal, and functional skills. Some of these skills are directly relevant to employment (e.g., use of public transportation to get to places, managing productive and adequate interpersonal interactions, and applying organization strategies [44]) or contribute in an indirect way through expanding the general experience of competence and awareness. The mutual contribution of different areas of life with a focus on occupational balance was previously demonstrated [45,46]. This notion placed doubt on the contribution of services segregated by areas of daily life functioning and highlighted the necessity to integrate vocational and other mental health services into an overall integrated intervention to promote the health and quality of life of young adults with SMI [46].

Contrary to the study hypothesis, no differences were found in motor skills between young adults with SMI who work and those who do not work. The lack of difference in motor skills between the groups in the present study may imply that in young adults motor functioning is a less restrictive factor for employment, in contrast to findings among the adult population with SMI [18]. This assumption is in line with an additional study that did not indicate the contribution of motor skills to success in employment [47]. An additional possible explanation for the little difference in motor skills as well as in cognitive skills among those receiving prevocational services and those who transferred to supported employment is an adaptation that was conducted through the job placement. The placement is based on personal skills and challenges and is performed through the interactive and continuous process of prevocational services followed by supported employment services. The holistic vision of the person, occupation, and environment using the scope of tools to evaluate a range of personal, environmental, and occupational factors and their impact on occupational performance and participation are of importance to lead the processes of employment opportunity identification and person-centered workplace adaptation [46,48,49]. 

## 5. Conclusions

To summarize, this study comprehensively investigated personal factors contributing to vocational outcomes in Israel with a unique focus on young adults with SMI. The results provided an initial understanding on the complex interplay between personal factors and demographic and work-related information to enable sustainable employment and highlighted the need for an integrated approach to enablers in the process of the vocational rehabilitation. We found that the interrelation between cognitive functioning, having a profession and work-related skills, and general independence in everyday life are of particular importance in the process of vocational rehabilitation. While investigating enablers for employment with retention, the study provided potential avenues for the promotion of vocational outcomes among young adults with SMI and may be informative for both people with SMI and policymakers for the planning and design of dedicated services. Obtaining a profession and work-related skills were found to be important enabling factors for sustainable employment in young adults, suggesting a need to invest resources in this field. Even though there is mounting evidence on the effectiveness of the “place and train” approach, the study alluded that the “train then place” approach may be useful to meet the unique needs of young adults with SMI in Israel and enable employment with retention. Moreover, the study implies that vocational rehabilitation should be conducted in the general context of individual life and occupations. Thus, additional implementation of the study results may represent a call for a reduction in the segregation of rehabilitation services by the areas of life activities and occupations, in order to encourage an integrative approach to rehabilitation and promote a personal journey of recovery. 

The study conclusions are limited due to methodological issues. The sample size was small and the participants were recruited from the only vocational rehabilitation facility for young adults; all of these issues affect the generalization of the study results. The study design was cross-sectional, limiting an in-depth understanding of the interplay between various enabling factors and their impact on vocational outcomes. Due to practical issues, some of the factors were assessed with screening tools, which may be less sensitive to individual differences. Given this, further research with longitudinal designs is needed to expand our understanding on the factors contributing to sustainable employment among young adults with SMI. In addition, future research should investigate the effectiveness of the implementation of an integrative approach for evaluation, which will include work-related self-efficacy, cognitive strategies, and level of independence in daily life for the enhancement of vocational outcomes among young adults with SMI in Israel. This research should be followed by studies investigating the contribution of various rehabilitation approaches and services to advance enablers in a valuable manner with regard to vocational outcomes. 

## Figures and Tables

**Table 1:** Comparing sample characteristics of both groups (continuous variables).

	SE (*N* = 21)			Prevocational Training (*N* = 21)			
	Range	M	SD	Range	M	SD	*t*
Years of education	9–16	11.8	1.3	9–17	11.6	1.5	−0.47
Years from illness onset	2–15	6.8	3	1–13	7	3.15	−0.53
Work hours per week	9.5–56	29.3	11.4	0	-	-	-
Time in current job (months)	2–24	7.3	5.6	0	-	-	-
Years of work experience	4–15	4.7	3.9	0–5	0.83	1.3	4.29 ***

Note: *** *p* < 0.001.

**Table 2:** Comparing sample characteristics between the two groups (categorical variables).

	SE (*N* = 21)		Prevocational Training (*N* = 21)		
	N	%	n	%	χ^2^
Living situation: outside the parental home	11	52.3	2	9.5	9.02 **
Having profession	10	47.6	1	4.8	9.98 **
Need help in IADL	4	19	10	47.6	3.86 *
Marital status (single)	19	90.5	21	100	2.1
Need help in housekeeping	6	28.6	8	31.8	0.43
Need emotional support	3	14.3	4	19	0.17
Need help in ADL	5	23.8	2	9.5	1.54

Note: * *p* < 0.05, ** *p* < 0.01.

**Table 3:** Work-related self-efficacy, cognitive skills, and motor skills among working and non-working participants.

		SE (*N* = 21)		Prevocational Training (*N* = 21)		Statistical Value
		SD	M	SD	M	
WSS-37	Career-planning skills	14.05	80.12	68.13	68.13	2.15 *^1^
	Job-securing skills	16.83	77.71	66.1	66.1	1.97 *^1^
	Work-related social skills	25.11	66.67	64.17	64.17	0.36 ^1^
	General work skills	10.57	86.37	74.7	74.7	2.39 *^1^
	Total score	12.08	79.8	69.56	69.56	2.06 *^1^
Lifting	Max. lift weight	7.26	12.57	12.38	12.38	−0.08 ^1^
	Max. lift number	1.11	3.14	3.05	3.05	−0.27 ^1^
PPT	Pins–dominant hand	2.85	12.38	12.67	12.67	0.35 ^1^
	Pins–non-dominant hand	2.38	11.52	11.81	11.81	0.39 ^1^
	Pair of pins	2.12	8.9	9.1	9.1	0.31 ^1^
Zoo test	Standard score	4.48	9.4	9.9	9.9	0.40 ^1^
	Total time, version 1 (seconds)	100.74	215.2	260.33	260.33	0.99 ^1^
	Total time, version 2 (seconds)	71.5	148.65	108.71	108.71	−2.01 *^1^
	Profile score	1.06	1.2	1.19	1.19	−0.25 ^2^

Note: * *p* < 0.05 (^1^ = *t*-test; ^2^ = Z Mann–Whitney); WSS-37—Work-related Self-Efficacy Scale, PPT—the Purdue Pegboard Test.

**Table 4:** Odds ratio for work integration.

	B	SE	OR ^§^	Wald
WSS: General work skills	0.17 *	0.069	1.186	6.04
Zoo test–version 2, total time (seconds)	0.02 *	0.01	1.02	4.34
Living situation	−3.96 **	1.49	0.02	7.02
Profession	5.67 **	2.26	290	6.28

* p < 0.05, ** *p* < 0.01, ^§^ Odds ratio.

## Data Availability

The data will be available upon the request from the corresponding author.
